# Another Boy Prodigy

**Published:** 1890-07

**Authors:** 


### ANOTHER BOY PRODIGY.

In Bridgeport, Conn., is a young Polish boy, named Paul Zilzisky, who has been performing wonders. His personal appearance is described as unprepossessing. He has a low forehead, with hair growing down to his eyebrows, is rather sleepy looking, and shambling in his movements. But whenever his father asks him a question relative to numbers, he at once brightens up and becomes excited. A writer in the Boston *Herald* says he met the boy and his father in a grocery store, and there witnessed illustrations of his power. The father said : "Paul, how many beans are there in this handful ?" The boy at once commenced to dance around the store, and became greatly excited as his father thrust his hand into a barrel of beans, took out a handful, and threw them down in a corner, where they lay scattered about. The lad leaped into the air, and almost before the beans touched the floor, shouted his reply. The beans were carefully gathered and counted, and while this was being done, the boy grinned and waited. The result showed him to be correct to a bean. He relapsed into his usual sleepy manner again. The father then seized a handful of oats, and put them in a heap upon the counter. "Paul," said he, "how many oats ?" The boy again jumped to his feet, devoured the oats mentally, and instantly shouted the number. It took a long time to count them, but the number was again found to be correct. Next the father seized a half-filled pail of water, and asked the boy, "How many cubic inches of water in the pail?" The boy sized it up with his eyes, and quickly shouted, "116." A careful computation proved the boy to be right. Other wonderful answers were given, always in an excited manner. After each and every successful answer, the same doleful expression returned.

